# A Protoberberine Derivative HWY336 Selectively Inhibits MKK4 and MKK7 in Mammalian Cells: The Importance of Activation Loop on Selectivity

**DOI:** 10.1371/journal.pone.0091037

**Published:** 2014-04-23

**Authors:** Namil Kim, Jeongyeon Park, Changdev G. Gadhe, Seung Joo Cho, Youngjin Oh, Donghyun Kim, Kiwon Song

**Affiliations:** 1 Department of Biochemistry, College of Life Science and Biotechnology, Yonsei University, Seoul, Korea; 2 Department of Bio-New Drug Development, College of Medicine, Chosun University, Gwangju, Korea; 3 Department of Cellular Molecular Medicine and Research Center for Resistant Cells, College of Medicine, Chosun University, Gwangju, Korea; 4 School of Electrical and Electronic Engineering, College of Engineering, Yonsei University, Seoul, Korea; National Cancer Institute, NIH, United States of America

## Abstract

A protoberberine derivative library was used to search for selective inhibitors against kinases of the mitogen-activated protein kinase (MAPK) cascades in mammalian cells. Among kinases in mammalian MAPK pathways, we identified a compound (HWY336) that selectively inhibits kinase activity of mitogen-activated protein kinase kinase 4 and 7 (MKK4 and MKK7). The IC_50_ of HWY336 was 6 µM for MKK4 and 10 µM for MKK7 *in vitro*. HWY336 bound to both kinases reversibly via noncovalent interactions, and inhibited their activity by interfering with access of a protein substrate to its binding site. The binding affinity of HWY336 to MKK4 was measured by surface plasmon resonance to determine a dissociation constant (*K_d_*) of 3.2 µM. When mammalian cells were treated with HWY336, MKK4 and MKK7 were selectively inhibited, resulting in inhibition of c-Jun NH_2_-terminal protein kinases *in vivo*. The structural model of HWY336 bound to either MKK4 or MKK7 predicted that HWY336 was docked to the activation loop, which is adjacent to the substrate binding site. This model suggested the importance of the activation loop of MKKs in HWY336 selectivity. We verified this model by mutating three critical residues within this loop of MKK4 to the corresponding residues in MKK3. The mutant MKK4 displayed similar kinase activity as wild-type kinase, but its activity was not inhibited by HWY336 compared to wild-type MKK4. We propose that the specific association of HWY336 to the activation loop of MKK4/MKK7 is responsible for its selective inhibition.

## Introduction

The evolutionarily conserved mitogen-activated protein kinase (MAPK) signaling pathway is activated by diverse extracellular stimuli to regulate proliferation, differentiation, and cell death in eukaryotes. The well-characterized MAPK family can be subdivided into three groups: c-Jun NH_2_-terminal protein kinases (JNKs), stress-activated protein kinases (SAPKs), p38, and extracellular signal-regulated kinases (ERKs). JNK/SAPKs are activated in response to chemical and environmental stresses through MKK4/MKK7. Studies have shown that inflammatory cytokines stimulate p38 MAPKs via MKK3/MKK6. Finally, ERKs are activated by mitogens and growth factors through sequential activation of MEKK Raf and MEK1/2 to control cell division and differentiation. Each MAPK pathway functions in parallel by mediating specific signals. Nevertheless, significant crosstalk exists between these pathways to affect the activation of a single MAPK and form a complex network [1]. Therefore, investigating how various signals are integrated to elicit stimulus- and tissue-specific responses is necessary to fully understand the mechanisms of MAPK signaling.

Synthetic inhibitors against specific components of MAPK cascades have been excellent tools for studying the mechanisms behind this signaling pathway in mammalian cells. For example, PD98059 [2-(2′-amino-3′-methoxyphenyl)-oxanaphthalen-4-one] and U0126 [1,4-diamino-2,3-dicyano-1,4-bis(2-aminophenylthio)butadiene] inhibit MEK1 and MEK2 [2], [3], respectively. In addition, the pyridinyl imidazoles SB203580 and SB202190 block SAPK2a/b and p38 MAPK isoforms, respectively [4]–[6], while the anthrapyrazole SP600125 [anthra[1,9-*cd*]pyrazol-6(2*H*)-one] inhibits JNK activity [7]. Because the MAPK signaling cascades control a variety of physiological processes directly, these selective inhibitors are currently being developed as possible pharmaceutical drugs [8]–[10].

Protoberberine alkaloids have anti-tumor activity in mice and cytotoxic activities against human cancer cell lines [11], [12]. In this study, we screened these compounds against kinases in the MAPK cascades using a protoberberine derivative library, and identified that a protoberberine derivative, HWY336, which selectively interacts and blocks MKK4/MKK7. Molecular modeling studies suggest that binding of HWY336 to the activation loop of MKK4 and MKK7 plays a major role in the inhibitory mechanism of this compound.

## Materials and Methods

### Cell culture

HEK293T and HEK293 cells were grown in DMEM (GIBCO) supplemented with 10% (v/v) fetal bovine serum (GIBCO), 100 U/ml penicillin, 100 µg/ml streptomycin, and 0.25 µg/ml amphotericin B (GIBCO). CHO cells were cultured in F-12K nutrient mixture medium (GIBCO) containing 10% FBS, 100 U/ml of penicillin, 100 µg/ml of streptomycin, and 0.25 µg/ml of amphotericin B (GIBCO). Cells were treated with 600 mM D-sorbitol (Sigma-Aldrich) for 30 min to activate MAPKs prior to purification.

### Kinase inhibitors

Each of 80 protoberberine derivatives was dissolved in DMSO to a final concentration of 1 mg/ml. A stock solution containing 1 mg/ml of SP600125, SB203580, or U0126 (A.G. Scientific Inc.) was also prepared in DMSO.

### Construction and expression of MKK4 and MKK7 mutant

The pCDNA3-Flag-MKK4-Q253Y I258V R262M vector and the pCDNA3-HA-MKK7-R283Y K288V R292M vector were created by site-directed mutagenesis with the pCDNA3-Flag-MKK4 vector (Addgene, Cambridge, MA) and the pCDNA3-HA-MKK7 vector (Dr. Chin Ha Chung, Seoul National University, Seoul, Korea) as a template. We mutated Gln to Tyr, Ile to Val, and Arg to Met in the MKK4. The following primers were used to amplify this mutant plasmid: 5′GGCATCAGTGGATACCTTGTGGACTCTGTGGCCAAGACAATGGATGCTGGCTGT3′ (forward),5′ACAGCCAGCATCCATTGTCTTGGCCACAGAGTCCACAAGGTATCCACTGATGCC3′ (reverse). Similarly, Arg to Tyr, Lys to Val, and Arg to Met in the MKK7 were mutated with the following primer: 5′GGCATCAGTGGATACCTTGTGGACTCTGTGGCCAAGACAATGTCAG CTGGCTGT3′(forward), 5′ACAGCCAGCTGACATTGTCTTGGCCACAGAGTCCACAAGGTA TCCACTGATGCC 3′ (reverse). The plasmid was treated with 10 U of DpnI (New England Biolabs, MA) for 1 hr after completion of PCR. The Q253Y I258V R262M mutations in MKK4 and R283Y K288V R292M mutations in MKK7 were confirmed by sequencing. Expression of MKK4, MKK4-Q253Y I258V R262M, MKK7, and MKK7-R283Y K288V R292M was confirmed by western blot using anti-FLAG M2 monoclonal antibody (Sigma-Aldrich) and HA probe (F-7) monoclonal antibody (Santa Cruz).

### Kinase immunoprecipitation and *in vitro* kinase assays

Cells were cultured on 100 mm plates for 2 days, scraped into 1.5-ml tubes, and collected by centrifugation at 2,000×*g* for 5 min at 4°C. The cell pellets were washed with cold PBS and solubilized with ice-cold 1× lysis buffer [20 mM Tris (pH 7.4), 150 mM NaCl, 1 mM EDTA, 1 mM EGTA, 1% Triton, 2.5 mM sodium pyrophosphate, 1 mM β-glycerolphosphate, 1 mM Na_3_VO_4_, and 1 µg/ml leupeptin]. Cellular extracts were centrifuged for 20 min at 10,000×*g* to remove cellular debris. The supernatant was used for immunoprecipitation. Cell lysate (∼500 µg total protein) was incubated overnight at 4°C with antibody specific to each kinase. The following antibodies were used: anti-MEK1 (rabbit polyclonal), anti-MEK2 (rabbit monoclonal), and anti-JNK (rabbit polyclonal) (Millipore, MA) at a 1∶200 dilution; anti-MKK3 (rabbit monoclonal), anti-MKK4 (rabbit polyclonal), anti-MKK6 (rabbit polyclonal), anti-MKK7 (rabbit polyclonal), anti-ERK1 (rabbit polyclonal), and anti-p38 (rabbit polyclonal) (New England Biolabs, Frankfurt, Germany) at a 1∶150 dilution. After the incubation, 60 µl Sepharose A-conjugated protein A (Sigma-Aldrich) was added and mixed for 90 min at 4°C. Immunoprecipitation of each kinase was confirmed by silver staining or western blot analysis.

For *in vitro* kinase assays, protein A bead-bound kinase was washed with cold kinase buffer [25 mM Tris (pH 7.5), 5 mM β-glycerolphosphate, 2 mM DTT, 0.1 mM Na_3_VO_4_, 10 mM MgCl_2_, and 1 mM PMSF], and incubated with HWY336, U0126, HWY289, berberine, or DMSO alone for 20 min at 30°C. The kinase reaction was conducted for 30 min at 30°C by addition of 0.5 µg JNK (Millipore) or 1 mg/ml myelin basic protein (MBP; Sigma-Aldrich), 1 mM ATP, and 10 µCi ^32^P-ATP (Perkin Elmer).

### High salt wash and competition assays

To determine whether HWY336 binding to MKK4 and MKK7 was reversible, each purified kinase was treated with HWY336 and washed three times with kinase buffer containing varying concentrations of NaCl from 0 to 500 mM prior to the kinase assay. Purified MKK4 and MKK7 were pre-incubated with 1 mg/ml MBP or 1 mM ATP prior to the addition of HWY336 to determine whether HWY336 competed with protein substrate or ATP for binding to MKK4 and MKK7.

### Western blot analysis

After HWY336 treatments, total protein (50 µg) of cell lysate was separated by 10% SDS-PAGE and transferred to a PVDF membrane. Specific proteins were detected using the following primary antibodies: anti-JNK, anti-p-JNKs, anti-p38, anti-p-p38, anti-MKK4, and anti-p-MKK4 (Cell Signaling Technology, Inc.).

### Surface plasmon resonance (SPR) measurement of the interaction between HWY336 and MKK4

The optical set-up for SPR detection consisted of two concentric dual motorized stages that were employed to implement angle-scanning capability. To measure binding constants, imaging SPR detection was performed by maintaining the angle of light incidence at 59°. Light source from a He-Ne laser (36 mW, λ = 632.8 nm, nominal beam diameter  = 650 µm, Melles-Griot, Carlsbad, CA) was p-polarized before incident on a SPR detection sample that was index-matched to an SF10 prism substrate. A p-i-n photodiode (818-UV, Newport) assessed the signal, which was subsequently fed to a low noise lock-in amplifier. The whole procedure was computer controlled and fully automated. Each measurement was repeated multiple times for statistical analysis.

SPR sample chips were fabricated by evaporating a 2 nm thick chromium adhesion layer and a 40 nm thick gold film on an SF10 cover glass substrate. Evaporated sample chips were cleaned in a plasma cleaner (Harrick Scientific Products, Pleasantville, New York). For SPR measurements, streptavidin was first coated on the gold surface by soaking the sample in a 1 mM solution of 8-amino-1-octanethiol (AOT, Dojindo) for 8 hrs in a dark environment and incubating the gold chip with 4 mM SATP solution for 1.5 hrs in a humid chamber. SPR sample surface was deacetylated in an incubation process using de-acetylation buffer (0.5 M hydroxylamine hydrochloride, 25 mM EDTA in PBS, pH 7.4) for 25 min, followed by streptavidin attachment on the surface using 20 µl of streptavidin-maleimide solution incubated on the SPR samples for 1 hr. After PBS washing, 10 nM biotinylated MKK4 solution (Biaffin GmbH & Co. KG, Kassel, Germany) was immobilized for 2 hrs. For binding of MKK4 with HWY336, HWY336 solution with PBS buffer was injected onto the SPR samples through a fluidic channel for 120 min using a micropump (KD Scientific).

### Homology modeling and molecular docking

Homology modeling for MKK4 was performed using the Modeller9v4 software that calculated a model composed of non-hydrogen atoms, based on aligning the sequence to be modeled with known related structures. A three-dimensional model was obtained by optimization of molecular probability density function using a variable target function procedure in Cartesian space that employed conjugate gradients and molecular dynamics with simulated annealing.

Molecular docking used a reported MKK7 structure and our homology model of MKK4 using the SYBYL 8.1 software package (Tripos, Inc., St. Louis, MO). Protein structure was prepared using a biopolymer module to prepare a structure that was suitable for docking assessments. Hydrogen atoms were added to the structure, atom types and charges were assigned using AMBER7 FF99, and side chain amides were modified. HWY336 was sketched using the SYBYL 8.1 sketch program and minimized using the Tripos force field and Powell method with a termination gradient set to 0.05 kcal/mol. The molecule was fully minimized with Gasteiger-Hückel charges. The docking study was performed using the Surflex-Dock module of SYBYL 8.1, which used an empirical scoring function to score ligand and protomol guided docking [13], [14].

## Results

### HWY336, a protoberberine derivative, was identified as a selective inhibitor of MKK4 and MKK7

**Figure 1:**
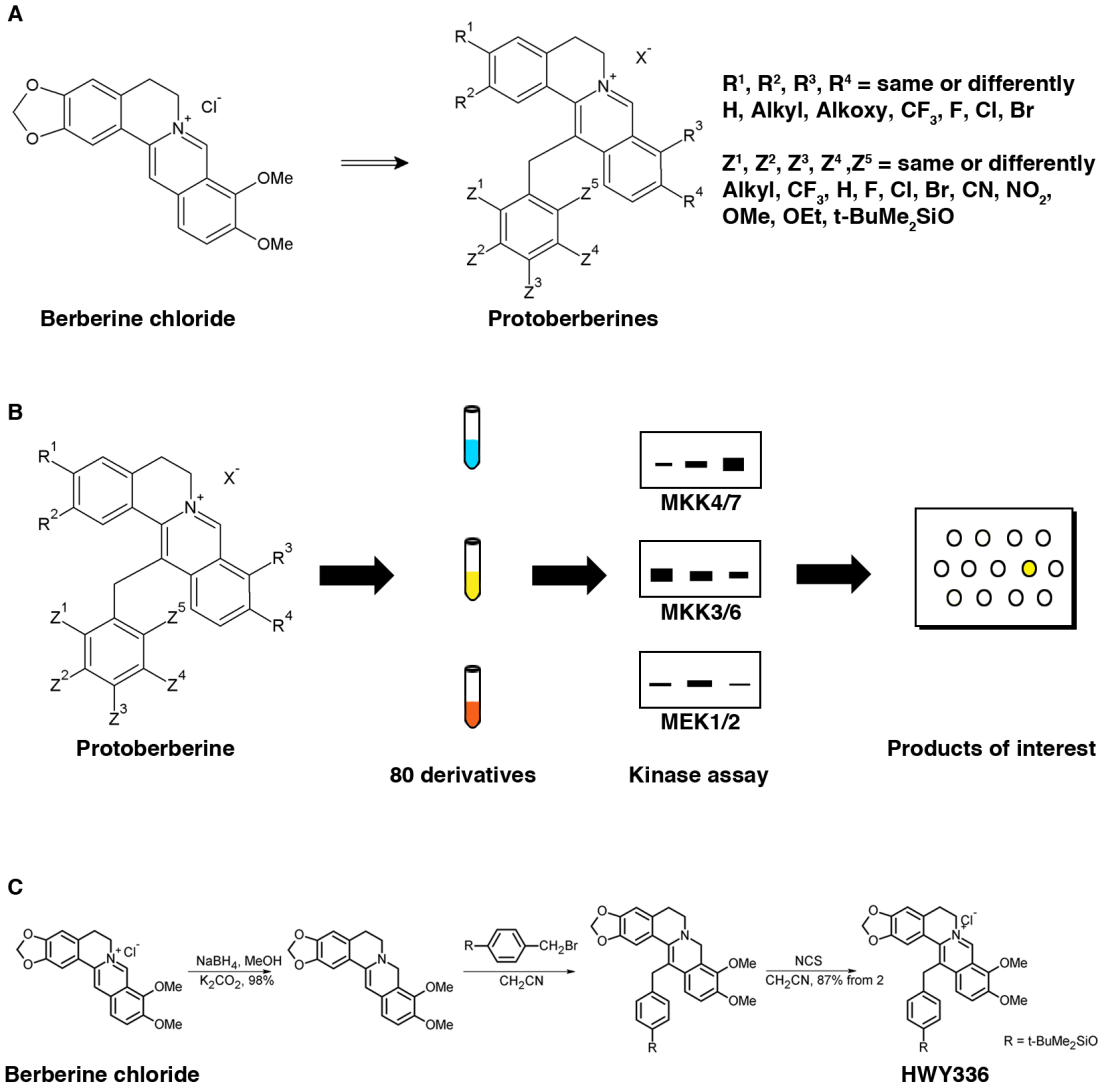
Identification of HWY336 as an inhibitor of MKK4 and MKK7 by screening a protoberberine derivative chemical library. A) Chemical modification of berberine chloride produced 80 protoberberine derivatives. B) Scheme for screening protoberberine derivatives for inhibition of various MKKs (MKK4/MKK7, MKK3/MKK6, and MEK1/MEK2) and MAPKs (JNKs, p38, and ERKs). Each MKK and MAPK was immunoprecipitated from HEK293 or CHO cells. C) Chemical structure and simplified synthetic process of HWY336 from berberine chloride.

A small-scale chemical library was generated by extensively modifying protoberberine (Figure 1A). To search for selective inhibitors of kinases in MAPK pathways using the library of 80 protoberberine derivatives, *in vitro* kinase assays were performed with MEK1, MEK2, MKK4, MKK7, MKK3, MKK6, and MAPKs (i.e., JNKs, p38, and ERKs) immunoprecipitated from either HEK293 or CHO cells, using MBP as a common substrate for kinases (Figure 1B). In this screen, we identified HWY336 as a selective inhibitor of MKK4 and MKK7 kinase activities (Figure S1 in File S1). The chemical structure of HWY336 and its synthesis from berberine chloride, the lead compound, are shown in Figure 1C.

As shown in Figure S1A, MKK4 and MKK7 kinase activity towards MBP was blocked completely by incubation with HWY336, while treatment with berberine chloride or another protoberberine derivative (HWY289) had no effect (Figure S1 in File S1). This selective inhibitory effect of HWY336 was observed regardless of the cell type from which MKK4 or MKK7 was purified (Figure S1A in File S1). Moreover, HWY336 inhibition of MKK4/MKK7 was comparable to that of the well-characterized MKK1/2 inhibitor U0126, which also blocks MKK4 and MKK7 at a higher concentration of 3 mM (Figure S1A in File S1).

### HWY336 selectively inhibits MKK4 and MKK7 *in vitro* and *in vivo*

**Figure 2:**
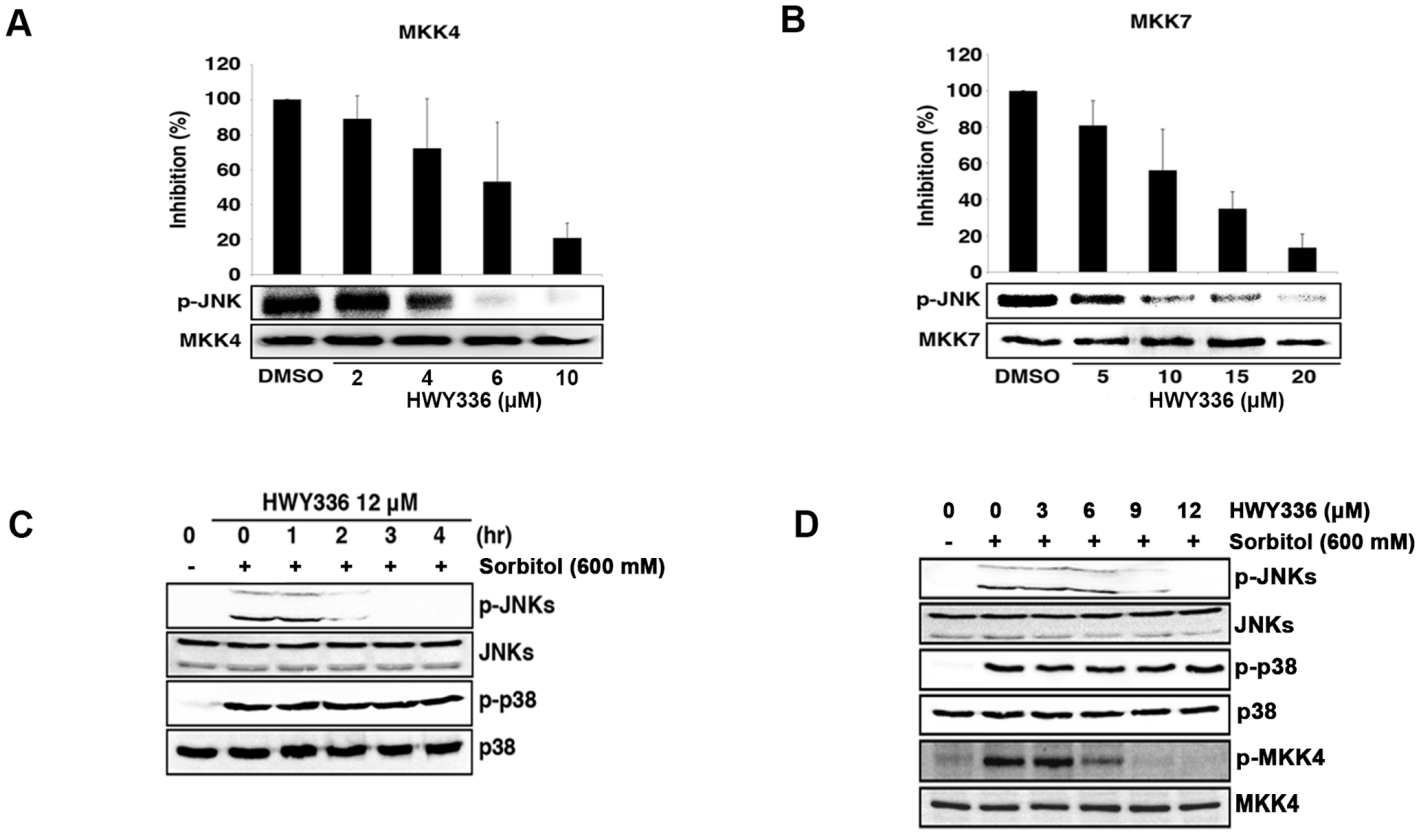
HWY336 inhibits MKK4 and MKK7 selectively in a concentration-dependent manner both *in vitro* and *in vivo*. A, B) MKK4 and MKK7 were immunoprecipitated from HEK293T cells following MAPK pathway activation as described in EXPERIMENTAL PROCEDURES. The same quantity of kinase was used in each assay. The activity of MKK4 (A) and MKK7 (B) was assayed by measuring γ-P^32^ phosphorylation of a endogenous substrate, JNK, in the presence of increasing HWY336 concentration and quantified using ImageJ software to determine IC_50_. The average relative kinase activity compared to DMSO is plotted as a percentage. Error bars represent the standard deviations. C, D) HEK293T cells were treated with 600 mM D-sorbitol for 30 min before harvest. (C) HEK293T cells were treated with 12 µM of HWY336 for 0, 1, 2, 3, or 4 hrs, and their total lysates were analyzed by western blots. p-JNKs, p-p38, JNKs, and p38 were detected as described. (D) HEK293T cells were treated with various concentrations of HWY336 (3, 6, 9, 12 µM) for 3 hrs and each cell lysate was prepared. p-JNKs, p-p38, p-MKK4, JNKs, p38, and MKK4 were detected by western blot analysis of cell lysates.

The discovery of HWY336-mediated inhibition of MKK4 and MKK7 from screening led us to identify HWY336 as a specific inhibitor of MKK4 and MKK7. However, MBP used as a substrate in the screen was not a physiological target for MKK4 and MKK7. Thus, we confirmed the selective inhibition of MKK4 and MKK7 by HWY336 with a physiological substrate, JNK. MKK4 kinase activity was completely inhibited by 10 µM HWY336, and MKK7 kinase activity was blocked by 20 µM HWY336. The IC_50_ of HWY336 was 6 µM for MKK4 (Figure 2A), and 10 µM for MKK7 (Figure 2B) *in vitro*. These data demonstrated that HWY336 inhibits MKK4/MKK7 kinase activity and blocks JNK phosphorylation.

To evaluate the selective inhibitory effect of HWY336 on the MAPK signaling pathway *in vivo*, we characterized the phosphorylation of endogenous JNKs and p38 by western blot analysis. Hyperosmotic stress triggers the activation of JNKs and/or p38 through their MAPK cascades in mammalian cells. We examined the effect of HWY336 on cells treated with D-sorbitol to activate JNK and p38 MAPK pathways in HEK293T cells. As shown in Figure 2C, treatment with 600 mM sorbitol led to the activation of JNKs and p38. A time course analysis revealed that JNK phosphorylation was decreased or disappeared while the amount of JNKs was not changed, when HEK293T cells were incubated in the presence of 12 µM HWY336 for 4 hrs (Figure 2C). When the sorbitol-treated cells were incubated with increasing concentrations of HWY336 for 3 hrs, phosphorylation of JNKs was reduced at 9 µM and disappeared at 12 µM of HWY336, but p38 phosphorylation was not affected (Figure 2D). Because MKK4/MKK7 becomes activated by phosphorylation, we also tested whether HWY336 inhibits the phosphorylation of MKK4 by examining the phosphorylation of MKK4 with anti-phospho MKK4 antibody. MKK4 phosphorylation is decreased when cells were treated with increasing concentrations of HWY336 (Figure 2D), demonstrating that HWY336 also blocks the phosphorylation of MKK4. These *in vivo* observations are consistent with the results of *in vitro* kinase assays in Figure 2A and B. Together, these results strongly suggest that HWY336 blocks the activation of JNKs by inhibiting upstream MKK4 and MKK7 both *in vitro* and *in vivo*.

### HWY336 directly binds to MKK4

When purified MKK4 or MKK7 was incubated with HWY336 and washed with kinase buffer containing varying concentrations of NaCl prior to the kinase assay, MKK4 and MKK7 activity was restored with increasing NaCl concentration (Figure S2A and S3A in File S1). These results suggested that the interaction of HWY336 to MKK4 and MKK7 was reversible and likely involved noncovalent interactions.

**Figure 3:**
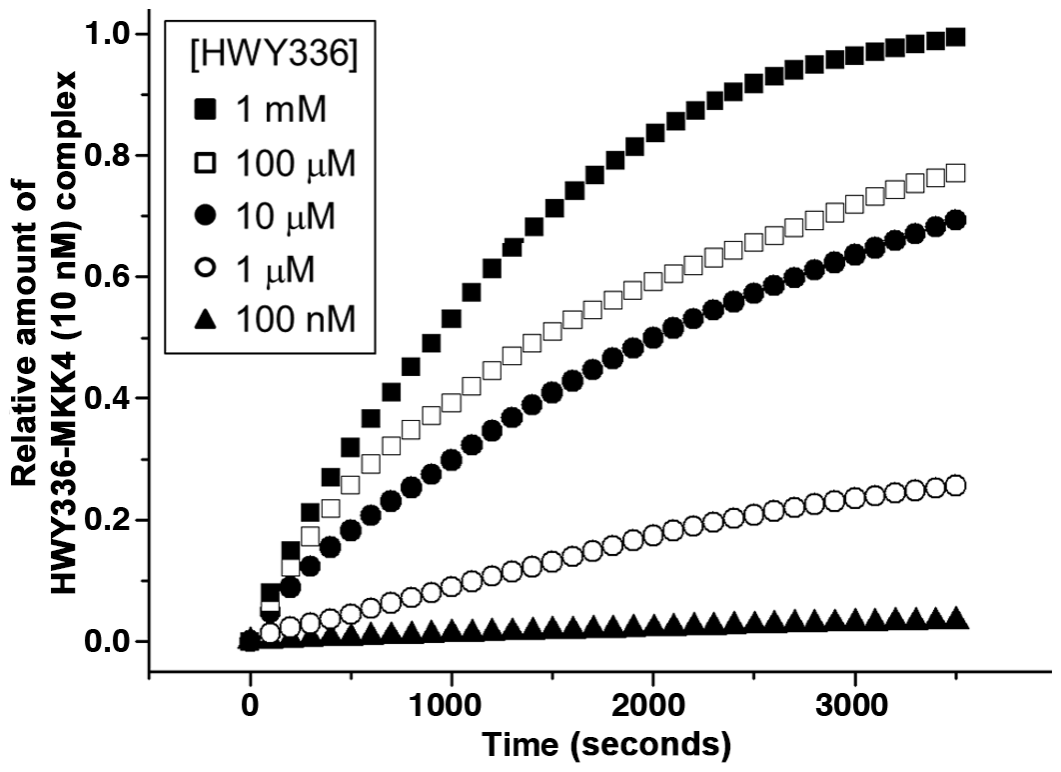
SPR detects the interaction of HWY336 with MKK4. The concentration of HWY336 was varied from 100-MKK4 complex with time was measured over the course of the interaction between HWY336 and MKK4.

To verify the binding affinity, we measured the interaction of MKK4 and HWY336 by SPR spectroscopy *in vitro*. SPR measurement was performed by injecting HWY336 through a microfluidic cell with varying concentrations from 100 nM to 1 mM. The concentration of MKK4 was fixed at 10 nM during immobilization onto the SPR sample surface. Figure 3 shows the relative amount of HWY336-MKK4 complex over time as a result of the interaction between HWY336 and MKK4. The relative amount of HWY336-MKK4 complex was assumed to be proportional to changes of light reflectance observed in SPR spectroscopy and defined here as the ratio of measured reflectance to the highest reflectance measured when the concentration of HWY336 was 1 mM. The results showed an increase amount of HWY336-MKK4 complex with time. In addition, the increase becomes greater as the concentration of HWY336 was increased.

The results in Figure 3 clearly confirmed the specific binding of HWY336 to MKK4, to produce an HWY336-MKK4 complex. The rate constant describing association of HWY336 and MKK4 was determined to be 66.31 M^−1^s^−1^. The dissociation constant (*K_d_*) of ligand-protein binding was 3.2 µM for MKK4 and HWY336, which was comparable to those of MEK1 and its well-known selective inhibitors, U0126 (*K*_d_ = 2±0.5 µM) and PD0325901 (*K*_d_ = 5±1 µM) [15].

### The activation loop of MKK4 and MKK7 is the HWY336 docking site

When we assessed whether HWY336 competed with a protein substrate or ATP for binding to MKK4 and MKK7, the inhibitory effect of HWY336 was unaffected by pre-incubation with ATP (Figure S2C and S3C in File S1), but was antagonized by MBP and JNK (Figure S2B and S3B in File S1). HWY336 no longer inhibited the kinase activity of MKK4 and MKK7 when MKK4 and MKK7 were pre-incubated with increasing concentrations of MBP and JNK (Figure S2B and S3B in File S1). These observations suggested that HWY336 either competes with a protein substrate for MKK4 and MKK7 binding, or at least blocks access of the substrate to its binding site.

**Figure 4:**
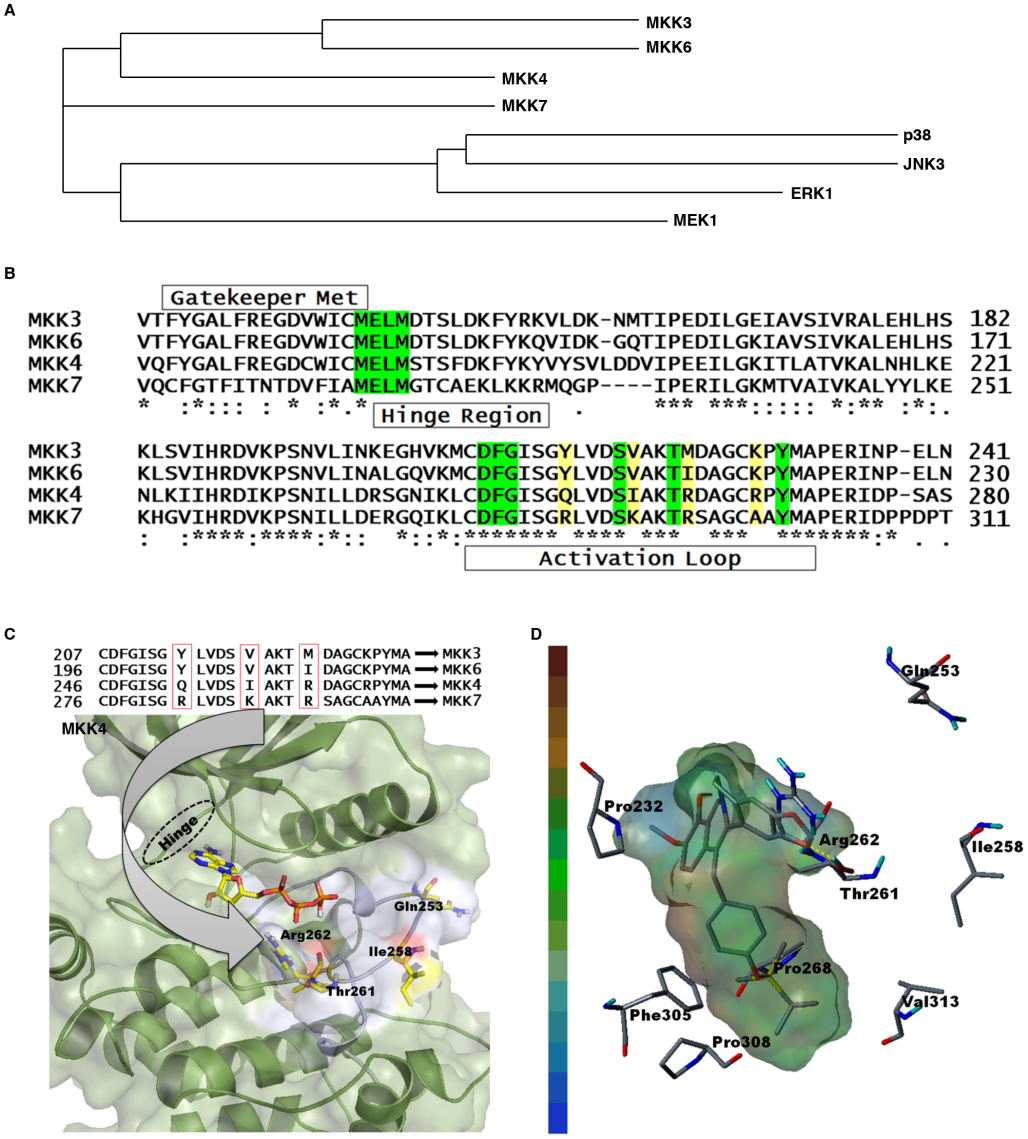
MKK4 and MKK7 homology in mammalian MAPK pathways. A) Phylogenetic tree for human MKK4, MKK7, MKK3, MKK6, MEK1, p38, JNK, and ERK. B) Amino acid sequence alignment starting from the hinge region to the activation loop are shown for MKK3 (UniProtKB accession code: P46734), MKK6 (UniProtKB accession code: P52564), MKK4 (UniProtKB accession code: P45985), and MKK7 (UniProtKB accession code: O14733). Green denotes a highly conserved region among these MKKs. Residues highlighted in yellow designate sequence variations in the activation loop. C, D) Three-dimensional structure of MKK4 suggests that HWY336 interacts with the activation loop through hydrogen bonding. C) (top) Amino acid sequence variations within the activation loop of MKKs. (bottom) Proposed docked pose of ATP in MKK4. The arrow designates different amino acids that may determine MKK selectivity. The MKK4 structure is shown in the background with the activation loop (white) containing the varying amino acids (Arg^262^) at the respective positions (generated with the Pymol program; www.pymol.org). D) Hydrophobic interactions between HWY336 and the MKK4 active site are shown. Hydrophobic residues within the active site are designated by the cap-stick model and HWY336 is displayed using transparent hydrophobic surfaces. The hydrophobicity index is displayed on the left, where brown and blue denote highly hydrophobic and hydrophilic areas, respectively. Pro^268^, Phe^305^, Pro^308^, and Val^313^ interact with HWY336 side chains. HWY336 interacts with the activation loop of MKK4 through hydrogen bonding via the hydroxyl group of Thr^261^.

To understand the basis of inhibitory selectivity of HWY336 to MKK4 and MKK7, we used molecular docking modeling of HWY336 and MKK4. As a first step in establishing a specific docking model for HWY336 binding to MKK4 and MKK7, the homology and phylogeny of these kinases were examined. In mammals, the MAPK cascades exist as two parallel pathways, MKK3/MKK6 and MKK4/MKK7 [16], [17]. Phylogenetic analysis based on sequence homology of p38, JNK3, ERK1, MEK1, MKK3/MKK6, and MKK4/MKK7 using ClustalW 2.0 (http://www.ebi.ac.uk/Tools/clustalw2/index.html) [18], demonstrated that MKK3/MKK6 and MKK4/MKK7 are more closely related to each other than to the other kinases (Figure 4A). Multiple sequence alignments of MKK4/MKK7 and MKK3/MKK6 using ClustalW revealed a gatekeeper hinge region, and an Asp-Phe-Gly (DFG) motif. Moreover, we identified highly conserved Ser, Thr, and Tyr residues within the activation loops of MKK4/MKK7 and MKK3/MKK6 (Figure 4B).

To further elucidate the inhibitory action of HWY336, we mapped its docking site by creating a three-dimensional model of MKK4 and MKK7. Since no crystal structure was currently available for MKK4, we used MKK7 (PDB code: 2DYL with resolution 2.45 Å) as a template for three-dimensional homology modeling for MKK4. MKK4 and MKK7 share 49% sequence identity. Therefore, the missing activation loop of MKK7 was first generated using the Modeller9v4 program [19]. A homology-based structure for MKK4 was modeled using the three-dimensional coordinates from the modified MKK7 template and Modeller9v4. The three-dimensional alignment between the MKK7 template and MKK4 structure is shown in Figure S4A in File S1, demonstrating that the three-dimensional structures of MKK4 and MKK7 are similar.

Molecular docking generates and scores putative protein-ligand complexes. Given the crystal structure of a binding target, molecular docking automatically samples ligand conformation and protein-ligand interactions with a specified region of the protein surface. This method has been used successfully to identify active compounds by filtering out those that do not fit into the binding site. Because our data demonstrated that HWY336 treatment leads to complete inhibition of MKK4/MKK7 activity but not MKK3/MKK6 (Figure S1B in File S1), we examined the three-dimensional selective docking of HWY336 to MKK4/MKK7 using the SYBYL 8.1 molecular modeling package installed on a Linux system. The structures of MKK4 and MKK7 were prepared using the biopolymer module of SYBYL 8.1.

The gatekeeper hinge region, DFG motif, and Ser/Thr and Tyr residues were highly conserved between MKK3/MKK6 and MKK4/MKK7 (Figure 4B). However, several sequence variations existed within the activation loop (Figure 4C). Therefore, we hypothesized that these varied residues may act as selectivity determinants among different MKKs. The active conformation in a number of kinases involves phosphorylation of conserved residues, either Ser/Thr or Tyr. Thus, phosphorylation of these key residues may shift the balance between active and inactive states. In highly active kinases, DFG motifs adopt a highly conserved “open conformation.”

We searched the possible ligand accessible cavities inside MKK4 as many as possible. Figure S5 in File S1 depicts the possible solvent pockets using SITE ID module of SYBYL 8.1.(Figure S5 in File S1). Site 1 is the ATP binding site and Site 2, the activation loop. Since HWY336 is a non-competitive inhibitor, ATP binding site was excluded. Among the remaining sites, only the site of activation loop has comparable size to HWY336. First, we docked ATP to the ATP-binding pocket and docked HWY336 near the activation loop. Based on the docking score, a docked pose was selected (Figure 4D).

HWY336 was positioned within the activation loop and near the phosphate binding region where the phosphate transformation process takes place. The docked pose for HWY336 in MKK4 showed hydrogen bonding with the conserved Thr^261^ residue, which is important for the phosphorylation process (Figure 4C). A similar docked conformation was observed for MKK7 (Figure S4B in File S1). In our model, HWY336 showed a similar binding mode in the activation loop of MKK4/MKK7. HWY336 possibly formed a hydrogen bond with Arg^262^ (Arg^292^ of MKK7) within the activation loops of MKK4 and MKK7, suggesting an important role of this amino acid residue. Hydrophobic interactions between HWY336 and MKK4/MKK7 were also important for its MKK inhibition. Hydrophobic interactions between HWY336 and MKK4 (Figure 4D) demonstrated that a HWY336 side chain docked to the hydrophobic pockets of Pro^268^, Phe^305^, Pro^308^, and Val^313^. In the MKK7 structure (Figure S4B in File S1), a HWY336 side chain interacted with the hydrophobic Ala^297^, Ala^298^, Phe^336^, and Val^347^ residues. The central 5-fused ring of HWY336 was positioned at the same location within MKK4 and MKK7, while groups attached to this ring had different orientations.

### Three residues within the MKK4/MKK7 activation loop were important for selective inhibition by HWY336

**Figure 5:**
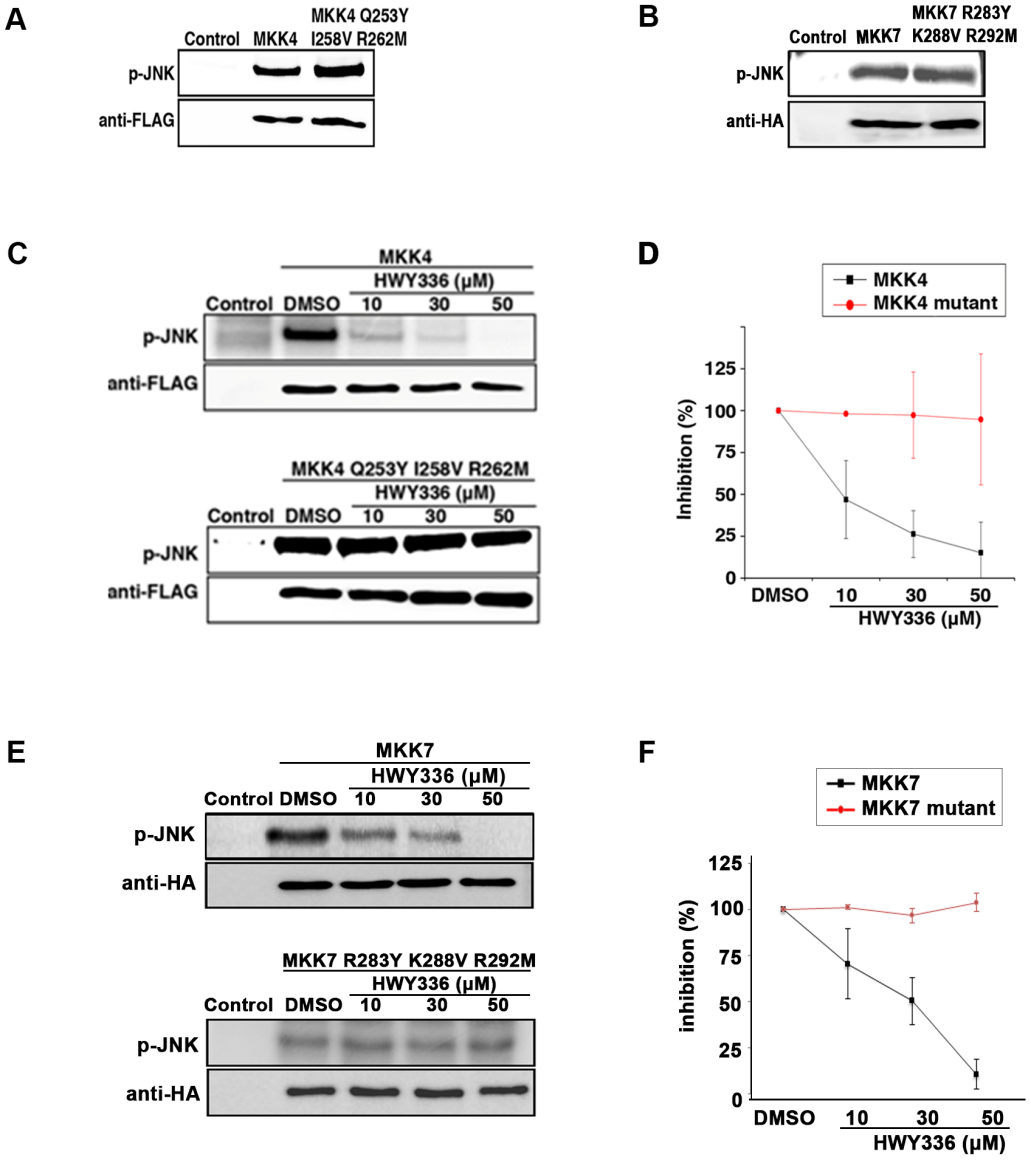
HWY336 does not inhibit the activity of MKK4 and MKK7 mutants of the proposed docking residues. Flag-tagged MKK4, MKK4-Q253Y I258V R262M, HA-tagged MKK7, and MKK7-R283Y K288V R292M were immunoprecipitated from activated HEK293 cells as described in Materials and Methods. Usage of the same amount of MKK4, MKK4 mutant, MKK7, and MKK7 mutant was confirmed by western blots with anti-flag antibody and anti-HA antibody, respectively. A, B) Kinase activity of immunoprecipitated (A) MKK4 and the MKK4 mutant, (B) MKK7 and the MKK7 mutant was assayed by measuring γ-P^32^ phosphorylation of JNK as a substrate. C–F) Effect of HWY336 on the kinase activity of (C, D) wild-type (top) and mutant (bottom) MKK4, (E, F) wild-type (top) and mutant (bottom) MKK7 was assayed after treatment with increasing concentrations of HWY336 (10, 30, 50 µM) or DMSO. D, F) Kinase activity of wild-type and mutant MKK4 and MKK7 with increasing concentrations of HWY336 shown in (C, E), was quantified by NIH ImageJ software.

Our structural model of MKK4 and MKK7 with HWY336 suggested that the activation loop was most likely a docking site for HWY336. Docking poses of HWY336 revealed similar binding between MKK4 and MKK7. The data also suggested that Gln^253^, Ile^258^, and Arg^262^ within MKK4, and Arg^283^, Lys^288^, and Arg^292^ within MKK7 differed from other highly conserved MKKs. Therefore, these activation loop residues were likely to be important for the selective docking of HWY336 (Figure 4C and Figure S4B in File S1). To confirm this hypothesis, we substituted Gln^253^, Ile^258^, and Arg^262^ of MKK4 and Arg^283^, Lys^288^, and Arg^292^ of MKK7 within the activation loop to Tyr, Val, and Met that correspond to residues in the activation loop of MKK3. MKK3, despite its homology to MKK4 and MKK7, was not susceptible to inhibition by HWY336. As expected, the mutants of activation loop, MKK4 mutant (Q253Y I258V R262M) and MKK7 mutant (R283Y K288V R292M), maintained similar kinase activity as the wild-type proteins because these residues were also present within the activation loop of MKK3 (Figure 5A and B).

Because these changes enabled retention of kinase activity (Figure 5A and B), we hypothesized that the MKK4 mutant (Q253Y I258V R262M) and MKK7 mutant (R283Y K288V R292M) would display reduced sensitivity to HWY336 if these residues were important for HWY336 docking. Thus, we evaluated the effect of HWY336 on the phosphorylation activity of both the MKK4 and MKK7 mutants on JNK. Incubation with 10 µM HWY336 reduced the kinase activity of wild-type MKK4 and MKK7 but did not affect the kinase activity of MKK4 and MKK7 mutants (Figure 5C, D, E, and F). Even after treatment with 50 µM HWY336, the kinase activity of both the MKK4 and MKK7 mutants was unaffected, while no kinase activity was observed in the wild-type (Figure 5C, D, E, and F). These results demonstrated that the three amino acid residues within the activation loop of MKK4 and MKK7 were important for selective inhibition of the kinase activity of MKK4 and MKK7 by HWY336. These observations strongly supported the roles of Gln^253^, Ile^258^, and Arg^262^ residues in MKK4, and Arg^283^, Lys^288^, and Arg^292^ of MKK7 during HWY336 docking.

## Discussion

In this study, we used a library of protoberberine derivatives to identify a selective inhibitor of the mammalian MAPK pathway. One of the compounds, HWY336, selectively inhibited MKK4 and MKK7 *in vitro* and *in vivo* by competing with protein substrates, suggesting that it associated with and/or modified the substrate binding site of MKK4 and MKK7. To understand the basis of selective inhibition of MKK4 and MKK7 by HWY336, we examined the direct binding of HWY336 with MKK4 by SPR and its docking with the predicted and established three-dimensional structures of MKK4 and MKK7. The three-dimensional modeling and docking studies suggested that HWY336 interacted with the activation loop of MKK4 and MKK7 adjacent to the substrate and ATP binding sites. Docking poses of HWY336 revealed similar binding between MKK4 and MKK7. Substituting the Gln^253^, Ile^258^, Arg^262^ of MKK4 and Arg^283^, Lys^288^, Arg^292^ of MKK7 within the activation loop to residues corresponding to those of MKK3 supported the possibility that the activation loop is a potential HWY336 docking site in MKK4 and MKK7. Moreover, we demonstrated that these conserved amino acids among MKK4 and MKK7 are important for selective inhibition of these kinases by HWY336.

Consistent with the inhibitory effect of HWY336 on mammalian MKK4 and MKK7, HWY336 also inhibited the stress-activated MAPKK Wis1 in *Schizosaccharomyces pombe* [20]. Wis1 is a homologue of mammalian MKK4 and MKK7 in *S. pombe*, suggesting a similarity in the three-dimensional structures of these proteins, even though they only share 41% and 38% sequence homology, respectively. Interestingly, their sequence identities were much higher within the activation loop, and 16 out of 26 amino acids were identical (Figure S5C in File S1). Moreover, the three critical residues within the activation loop of MKK4 (Gln^253^, Ile^258^, and Arg^262^) had corresponding counterparts in *S. pombe* Wis1 (Asn^465^, Ile^470^, and Asn^474^; Figure S5C in File S1). Because Gln^253^ and Asn^465^ possessed an amide group as a side chain, other differences contributed by these residues most likely had a minimal effect on activation. The chemical property of Asn^474^ in Wis1, which corresponded to Arg^262^ in MKK4, contributed the greatest differences. While Asn is polar and uncharged, Arg is polar and basic. However, because these residues contain polar side chains, the change from Arg to Asn could be regarded as functionally conserved. The terminal guanidinium group of Arg^262^ in MKK4 acts as a hydrogen bond donor in our structural modeling study. We would expect that the terminal amide group of the Asn^474^ residue of Wis1 could also function as a hydrogen donor when it binds to HWY336.

The binding poses of ATP and HWY336 in our model indicated that HWY336 is a non-ATP competitive inhibitor of MKK4/MKK7. Our docking models strongly suggested that HWY336 inhibited MKK4/MKK7 phosphorylation within the activation loop or hindered substrate access to these protein kinases. Additionally, hydrogen bonding interactions between HWY336 and MKK4/MKK7 along with a washing experiment (Figure S2A and S3A in File S1) demonstrated that this compound bound reversibly and noncovalently. In general, phosphorylation at specific residues (Ser/Thr and Tyr) within the activation loop functions as a molecular switch for cellular communication. The phosphorylation process induces conformational changes such that the position of the highly conserved activation loop is altered to allow access to the substrate-binding domain. Thus, differences in the conformational state of the activation loop may play a major role in signaling specificity of each MAPKK in response to various stresses.

Our finding that HWY336 specifically inhibited the kinase activity of mammalian MKK4 and MKK7 *in vitro* and *in vivo* is significant, because no selective inhibitor has yet been reported or developed for these kinases. Although much effort has been devoted to developing selective inhibitors for each kinase in the mammalian MAPK pathway, specificity has been a major challenge. Some successful approaches targeted the ATP-binding site. For example, SB203580 and SP600125 blocked p38 and JNK kinase activity, respectively, by interacting with the ATP-binding domain [5]–[7]. Alternatively, several non-ATP competitive inhibitors have also been developed, providing the opportunity to differentially target a specific component of the MAPK signal transduction cascade. PD98059 was the first specific MEK1/2 inhibitor that acted by binding to the inactive conformation of the enzyme to prevent its activation by Raf [2], [3]. This inhibitor has also served as a standard pharmacologic tool for cellular studies of the ERK MAPK pathway. U0126, another MEK1/2 inhibitor, is also non-ATP competitive compound and is more potent than PD98059 [3].

In the present report, we have shown that HWY336 reversibly inhibited MMK4 and MKK7 by competing with protein substrates. HWY336 was of particular interest because its mechanism of inhibition is novel. Because kinases limit their substrates through specificity, and HWY336 competes with substrate(s) for binding, further study on the mechanism underlying the effects of HWY336 would provide important information that could be used to overcome the specificity barrier in the development of MAPK inhibitors. In addition, the high selectivity of HWY336 for MKK4 and MKK7 suggests that further modification of these compounds may lead to the development of selective inhibitors as therapeutics.

## Supporting Information

File S1 **Supporting information figures.** **Figure S1**, **HWY336 inhibits MKK4 and MKK7 selectively but does not inhibit other mammalian MEKs and MAPKs.** MKK4 and MKK7 were immunoprecipitated from HEK293 or CHO cells following MAPK pathway activation as described in EXPERIMENTAL PROCEDURES. The same quantity of kinase was used in each assay. A) MKK4 and MKK7 activity assayed with MBP as a substrate after pre-treatment with 1 mM of DMSO (lane 1), 400 µM berberine (lane 2), 1 mM HWY289, another protoberberine derivative from the library (lane 4), or 1 mM HWY336 (lane 5). DMSO was used as a negative control (lane 1) and 3 mM U0126 was used as a positive control (lane 3). B, berberine; U, U0126; 289, HWY289; 336, HWY336. B) MEK1, MKK3/MKK6, JNK1/2, p38, and ERK1/2 were immunoprecipitated from activated HEK293 and CHO cells as described in Materials and Methods. The activity of each immunoprecipitated kinase was assayed using MBP as a substrate after pre-treatment with 1 mM DMSO (lane 1), 400 µM berberine (lane 2), 1 mM HWY289 (lane 4), or 1 mM HWY336 (lane 5). Established inhibitors against each kinase (U0126 25 µM for MEK1, 3 mM for MKK3/MKK6, ERK1/2, and SP600125, 25 µM for JNK1/2 and SB203580, 25 µM for p38) were used as positive controls (lane 3). An equal amount of each kinase was used in all assays. B, Berberine; SP, SP600125; SB, SB203580; U, U0126; 289, HWY289; 336, HWY336. **Figure S2, The mechanism of HWY336-mediated inhibition of MKK4 and MKK7.** Stress-activated MKK4 and MKK7 were immunoprecipitated from HEK293 cells, and the same amount of each kinase was used in the assays as confirmed by Coomassie staining or western blotting. A) Reversibility of HWY336 binding to MKK4 and MKK7. MKK4 or MKK7 was pre-incubated with 600 µM HWY336 for 10 min and washed with kinase buffer containing increasing concentrations of NaCl prior to kinase assays. B, C) The ability of ATP (B) or MBP substrate (C) to compete with HWY336 for binding to MKK4 and MKK7. Immunoprecipitated MKK4 and MKK7 were pre-incubated with different concentrations of either ATP (B) or MBP (C) and their kinase activities were assayed in the presence of 600 µM HWY336 or DMSO (lane 1). **Figure S3, HWY336 interacts with MKK4 reversibly, and inhibits MKK4 by competing with JNK, a physiological protein substrate, and not with ATP.** Stress-activated MKK4 were immunoprecipitated from HEK293 cells as described in Materials and Methods. A) HWY336 interacts with MKK4 reversibly. Immunoprecipitated MKK4 was treated with 50 µM HWY336 for 10 min and washed with kinase buffer containing different concentrations of NaCl as in Figure S2A, B, C of Supplemental Figures. Competition of (B) ATP or (C) JNK substrate with HWY336 for binding to MKK4. MKK4 was immunopreciptiated and pre-incubated with different concentrations of either (B) ATP (C) or JNK, and their kinase activities were assayed in the presence of 50 µM HWY336 or DMSO (lane 1). **Figure S4**, **Computer modeling predicts the conserved three-dimensional structures of MKK4 over MKK7.** A) Three-dimensional structure of MKK7 (magenta) is superimposed onto the MKK4 (cyan) model structure. B) Three-dimensional modeling of HWY336 docking with MKK7. A proposed docked pose of ATP inside the MKK7 structure. The residues that vary (Arg^283^, Lys^288^, Thr^291^ and Arg^292^) within the activation loop are indicated. HWY336 forms a hydrogen bond with Arg^292^ and the conserved Thr^291^, which is important in the phosphorylation process of MKK7 (generated with the Pymol program; www.pymol.org). C) Activation loop sequences and critical residues are highly conserved among MKK4/MKK7 and Wis1, which are all inhibited by HWY336. **Figure S5, Prediction of a HWY336-binding site within the activation loop of MKK4.** All possible solvent pockets were simulated and a HWY336-binding site was predicted within the activation loop of MKK4 using SITE ID module of SYBYL 8.1.
(PDF)
