# Supplementary material for: A Protoberberine Derivative HWY336 Selectively Inhibits MKK4 and MKK7 in Mammalian Cells: The Importance of Activation Loop on Selectivity
Source: PLoS One. 2014 Apr 23;9(4):e91037. doi: 10.1371/journal.pone.0091037 (PMC3997336; doi:10.1371/journal.pone.0091037)
Supplement: File S1 — Supporting information figures. Figure S1, HWY336 inhibits MKK4 and MKK7 selectively but does not inhibit other mammalian MEKs and MAPKs. MKK4 and MKK7 were immunoprecipitated from HEK293 or CHO cells following MAPK pathway activation as described in EXPERIMENTAL PROCEDURES. The same quantity of kinase was used in each assay. A) MKK4 and MKK7 activity assayed with MBP as a substrate after pre-treatment with 1 mM of DMSO (lane 1), 400 µM berberine (lane 2), 1 mM HWY289, another protoberberine derivative from the library (lane 4), or 1 mM HWY336 (lane 5). DMSO was used as a negative control (lane 1) and 3 mM U0126 was used as a positive control (lane 3). B, berberine; U, U0126; 289, HWY289; 336, HWY336. B) MEK1, MKK3/MKK6, JNK1/2, p38, and ERK1/2 were immunoprecipitated from activated HEK293 and CHO cells as described in Materials and Methods. The activity of each immunoprecipitated kinase was assayed using MBP as a substrate after pre-treatment with 1 mM DMSO (lane 1), 400 µM berberine (lane 2), 1 mM HWY289 (lane 4), or 1 mM HWY336 (lane 5). Established inhibitors against each kinase (U0126 25 µM for MEK1, 3 mM for MKK3/MKK6, ERK1/2, and SP600125, 25 µM for JNK1/2 and SB203580, 25 µM for p38) were used as positive controls (lane 3). An equal amount of each kinase was used in all assays. B, Berberine; SP, SP600125; SB, SB203580; U, U0126; 289, HWY289; 336, HWY336. Figure S2, The mechanism of HWY336-mediated inhibition of MKK4 and MKK7. Stress-activated MKK4 and MKK7 were immunoprecipitated from HEK293 cells, and the same amount of each kinase was used in the assays as confirmed by Coomassie staining or western blotting. A) Reversibility of HWY336 binding to MKK4 and MKK7. MKK4 or MKK7 was pre-incubated with 600 µM HWY336 for 10 min and washed with kinase buffer containing increasing concentrations of NaCl prior to kinase assays. B, C) The ability of ATP (B) or MBP substrate (C) to compete with HWY336 for binding to MKK4 and MKK7. Immunoprecipitated MKK4 and MKK7 were [file pone.0091037.s001.pdf]

Figure S1

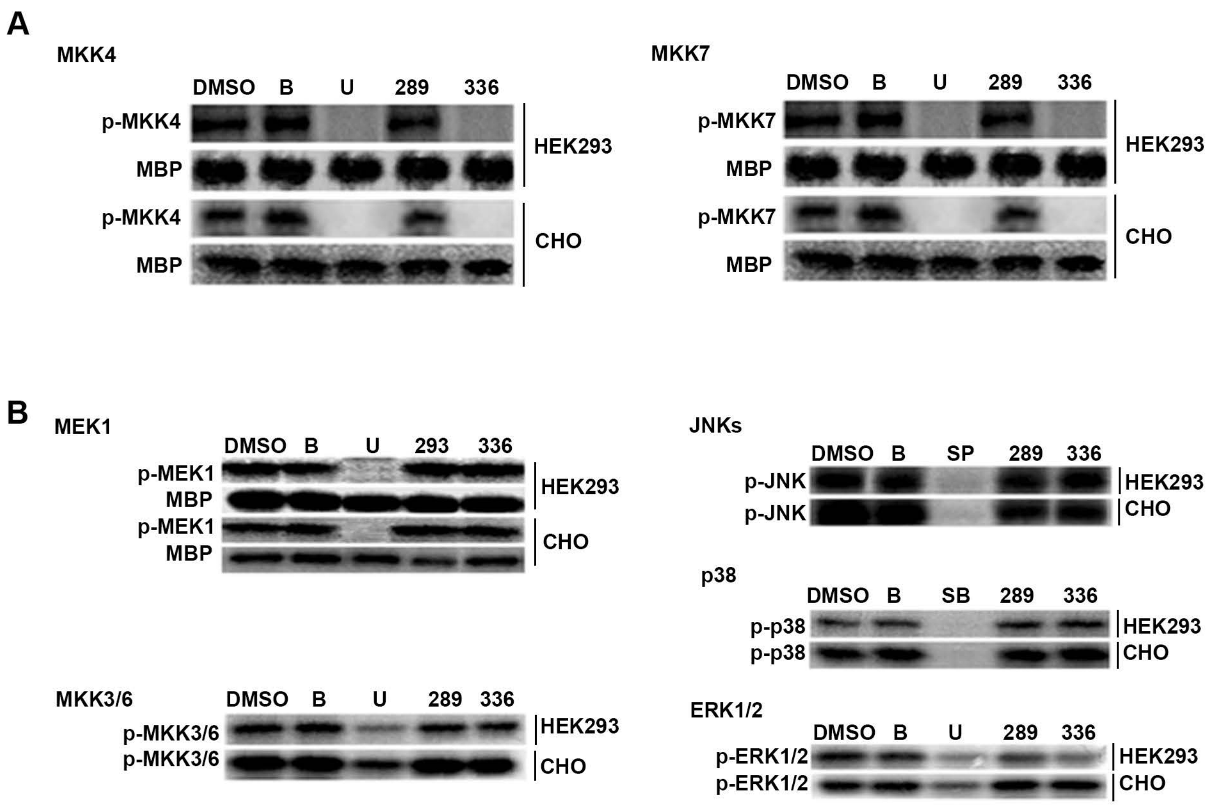
A
MKK4
DMSO B U 289 336
p-MKK4
MBP
p-MKK4
MBP
HEK293
CHO
MKK7
DMSO B U 289 336
p-MKK7
MBP
p-MKK7
MBP
HEK293
CHO
B
MEK1
DMSO B U 293 336
p-MEK1
MBP
p-MEK1
MBP
HEK293
CHO
JNKs
DMSO B SP 289 336
p-JNK
p-JNK
HEK293
CHO
p38
DMSO B SB 289 336
p-p38
p-p38
HEK293
CHO
MKK3/6
DMSO B U 289 336
p-MKK3/6
p-MKK3/6
HEK293
CHO
ERK1/2
DMSO B U 289 336
p-ERK1/2
p-ERK1/2
HEK293
CHO

Figure S2

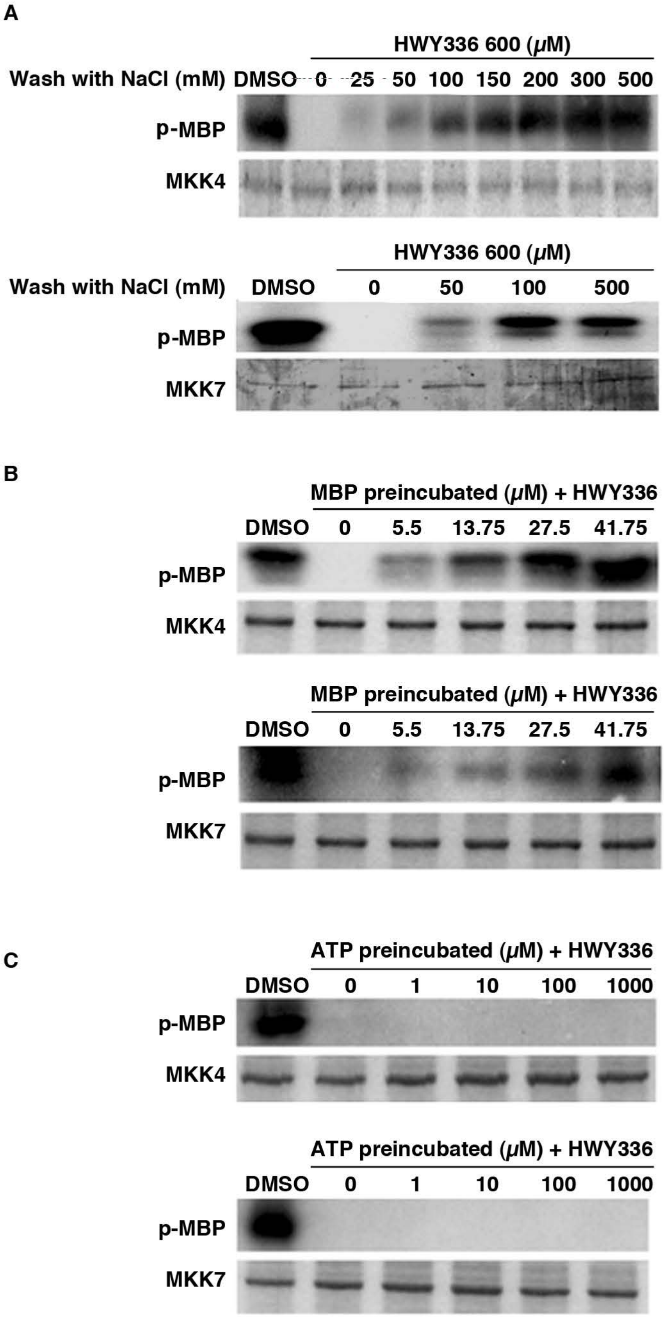

A
HWY336 600 (μM)
Wash with NaCl (mM) DMSO 0 25 50 100 150 200 300 500
p-MBP
MKK4
HWY336 600 (μM)
Wash with NaCl (mM) DMSO 0 50 100 500
p-MBP
MKK7
B
MBP preincubated (μM) + HWY336
DMSO 0 5.5 13.75 27.5 41.75
p-MBP
MKK4
MBP preincubated (μM) + HWY336
DMSO 0 5.5 13.75 27.5 41.75
p-MBP
MKK7
C
ATP preincubated (μM) + HWY336
DMSO 0 1 10 100 1000
p-MBP
MKK4
ATP preincubated (μM) + HWY336
DMSO 0 1 10 100 1000
p-MBP
MKK7

Figure S3

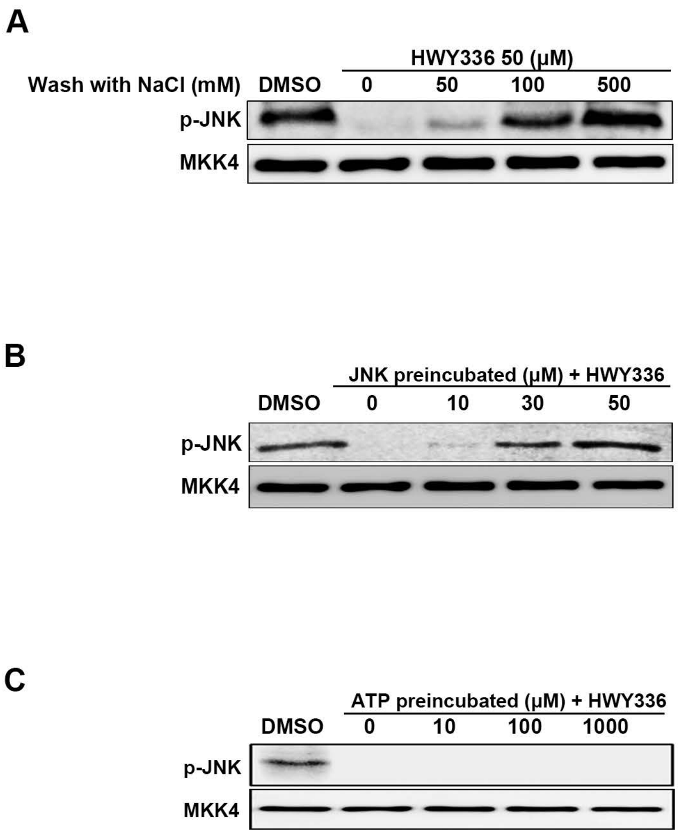
A
HWY336 50 (μM)
Wash with NaCl (mM)
DMSO
0
50
100
500
p-JNK
MKK4
B
JNK preincubated (μM) + HWY336
DMSO
0
10
30
50
p-JNK
MKK4
C
ATP preincubated (μM) + HWY336
DMSO
0
10
100
1000
p-JNK
MKK4

Figure S4

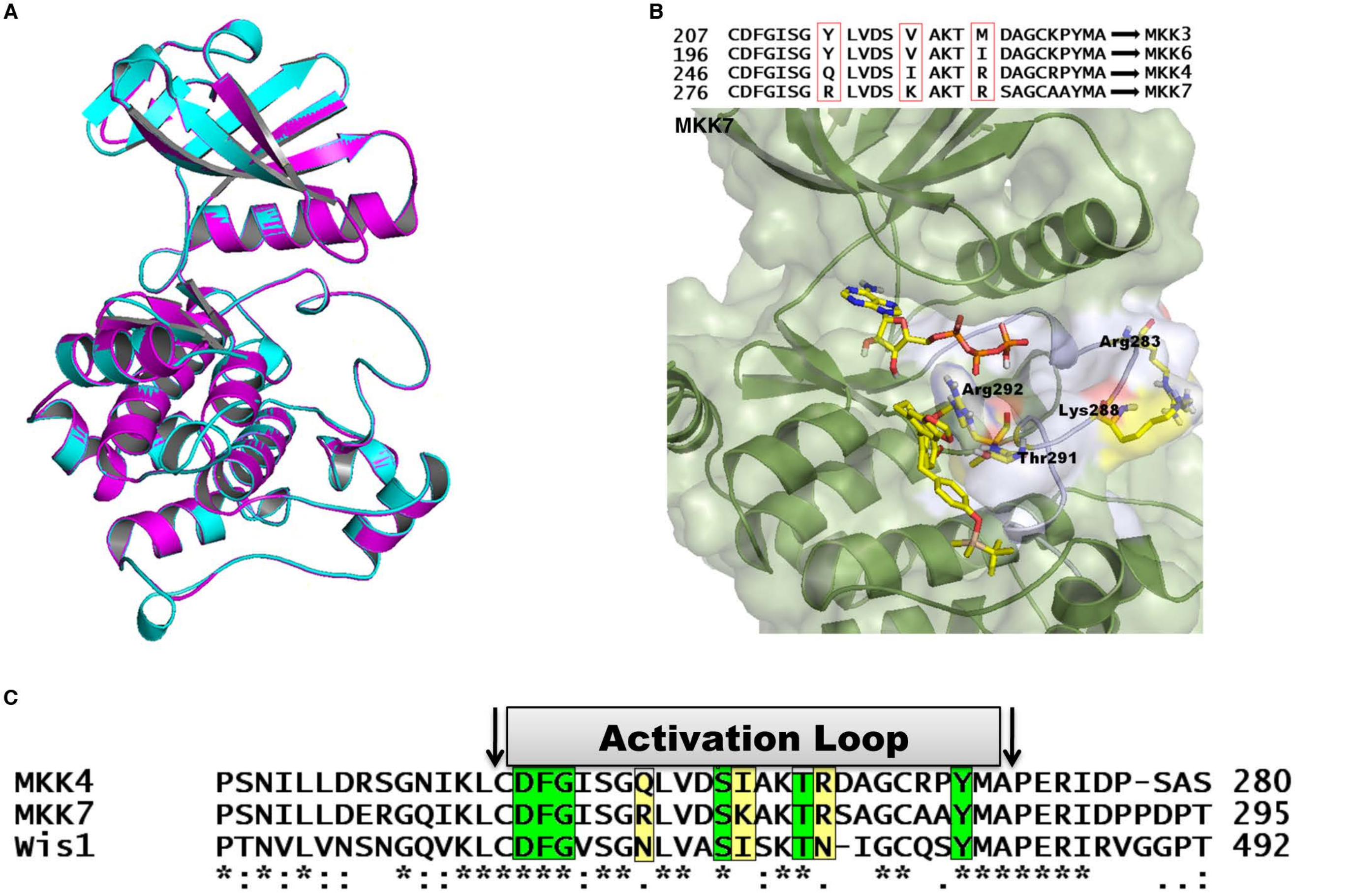
A
B
207 CDFGISG Y LVDS V AKT M DAGCKPYMA → MKK3
196 CDFGISG Y LVDS V AKT I DAGCKPYMA → MKK6
246 CDFGISG Q LVDS I AKT R DAGCRPYMA → MKK4
276 CDFGISG R LVDS K AKT R SAGCAAYMA → MKK7
MKK7
Arg283
Arg292
Lys288
Thr291
C
Activation Loop
MKK4 PSNILLDRSGNIKLCDFGISGQLVDSIAKTRDAGCRPYMAPERIDP-SAS 280
MKK7 PSNILLDERGQIKLCDFGISGRLVDSKAKTRSAGCAAYMAPERIDPPDPT 295
Wis1 PTNVLVNSNGQVKLCDFGVSGNLVASISKTN-IGCQSYMAPERIRVGGPT 492

Figure S5

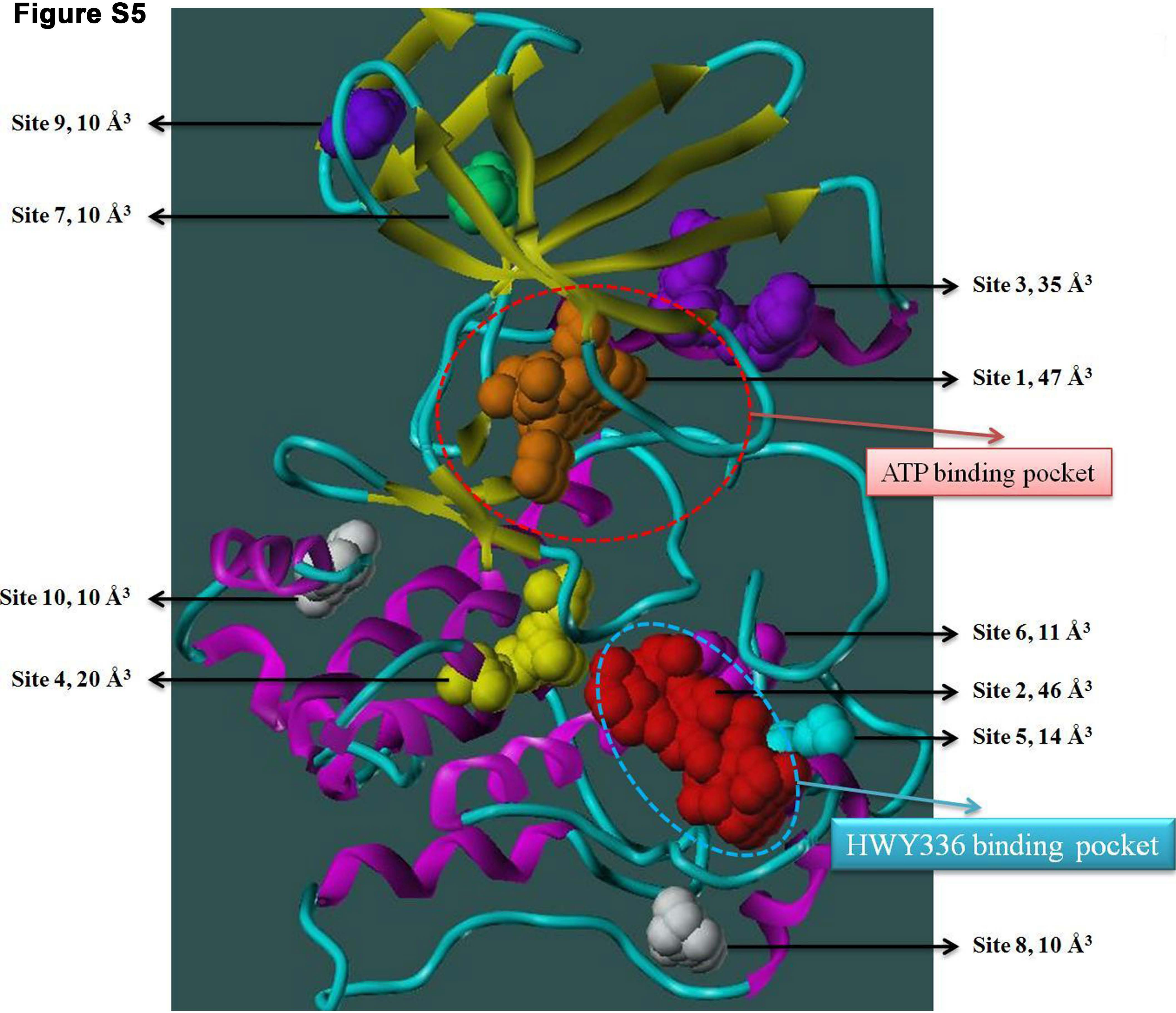

Site 9, 10 Å³
Site 7, 10 Å³
Site 3, 35 Å³
Site 1, 47 Å³
ATP binding pocket
Site 10, 10 Å³
Site 6, 11 Å³
Site 4, 20 Å³
Site 2, 46 Å³
Site 5, 14 Å³
HWY336 binding pocket
Site 8, 10 Å³
